# Chronic circadian desynchronization of feeding-fasting rhythm generates alterations in daily glycemia, LDL cholesterolemia and microbiota composition in mice

**DOI:** 10.3389/fnut.2023.1154647

**Published:** 2023-04-14

**Authors:** Laura Lucía Trebucq, Melisa Luciana Lamberti, Rosana Rota, Ignacio Aiello, Cristina Borio, Marcos Bilen, Diego Andrés Golombek, Santiago Andrés Plano, Juan José Chiesa

**Affiliations:** ^1^Laboratorio de Cronobiología, Departamento de Ciencia y Tecnología, Universidad Nacional de Quilmes (UNQ), Consejo Nacional de Investigaciones Científicas y Técnicas (CONICET), Bernal, Argentina; ^2^Laboratorio de Ingeniería Genética, Biología Celular y Molecular, Departamento de Ciencia y Tecnología, Universidad Nacional de Quilmes (UNQ), Consejo Nacional de Investigaciones Científicas y Técnicas (CONICET), Bernal, Argentina; ^3^Escuela de Educacion, Universidad de San Andrés, Victoria, Argentina; ^4^Institute for Biomedical Research (BIOMED), Catholic University of Argentina (UCA), National Scientific and Technical Research Council (CONICET), Buenos Aires, Argentina

**Keywords:** chronic jetlag, hyperinsulinemia, circadian rhythm, glucose intolerance, lipidemia

## Abstract

**Introduction:**

The circadian system synchronizes behavior and physiology to the 24-h light– dark (LD) cycle. Timing of food intake and fasting periods provide strong signals for peripheral circadian clocks regulating nutrient assimilation, glucose, and lipid metabolism. Mice under 12 h light:12 h dark (LD) cycles exhibit behavioral activity and feeding during the dark period, while fasting occurs at rest during light. Disruption of energy metabolism, leading to an increase in body mass, was reported in experimental models of circadian desynchronization. In this work, the effects of chronic advances of the LD cycles (chronic jet-lag protocol, CJL) were studied on the daily homeostasis of energy metabolism and weight gain.

**Methods:**

Male C57 mice were subjected to a CJL or LD schedule, measuring IPGTT, insulinemia, microbiome composition and lipidemia.

**Results:**

Mice under CJL show behavioral desynchronization and feeding activity distributed similarly at the light and dark hours and, although feeding a similar daily amount of food as compared to controls, show an increase in weight gain. In addition, ad libitum glycemia rhythm was abolished in CJL-subjected mice, showing similar blood glucose values at light and dark. CJL also generated glucose intolerance at dark in an intraperitoneal glucose tolerance test (IPGTT), with increased insulin release at both light and dark periods. Low-density lipoprotein (LDL) cholesterolemia was increased under this condition, but no changes in HDL cholesterolemia were observed. Firmicutes/Bacteroidetes ratio was analyzed as a marker of circadian disruption of microbiota composition, showing opposite phases at the light and dark when comparing LD vs. CJL.

**Discussion:**

Chronic misalignment of feeding/fasting rhythm leads to metabolic disturbances generating nocturnal hyperglycemia, glucose intolerance and hyperinsulinemia in a IPGTT, increased LDL cholesterolemia, and increased weight gain, underscoring the importance of the timing of food consumption with respect to the circadian system for metabolic health.

## 1. Introduction

The circadian clock, located at the hypothalamic suprachiasmatic nucleus (SCN), consists of neuronal network oscillators based on transcription-translation feedback loops of core clock genes, whose activity is synchronized to the 24-h LD cycle through light-activated pathways (1). Briefly, the CLOCK and BMAL1 heterodimer bind to the E-boxes of their own repressors Cryptochrome (Cry1 and Cry2) and Period (Per1, 2, and 3) and of the nuclear hormone receptors Rev-erb (α and β), and Ror (α, β, and γ). In turn, this molecular clockwork is fine-tuned by ROR activation and REV-ERB repression of the Bmal1 gene expression through RRE elements (2). Outputs from the SCN relaying in several hypothalamic areas generate daily activity-rest, endocrine, physiological, and metabolic rhythms. In virtue of similar molecular machinery, peripheral clocks coordinate circadian metabolic functions downstream of the SCN (3). Feedback signals from the feeding-fasting rhythm act as strong zeitgebers (synchronizers) for oscillators in metabolic tissues controlling energy homeostasis, indeed bypassing the SCN under time-restricted feeding protocols (4), or without its participation (5). In fact, the liver clock is a central circadian integrator of feeding signals regulating rhythmic transcripts involved in daily carbohydrate homeostasis (6).

Both diurnal and nocturnal species feed when they are behaviorally active, concurrent with a high metabolic and thermogenic rate. Mice synchronized to laboratory LD cycles show nocturnal activity with about 75–85% of caloric consumption occurring at dark hours (7). This ensures the supply of carbohydrates, lipids, and amino acids needed for energy uptake, utilization, and storage, while basal energy expenditure during the rest/fasting period is sustained by the degradation and mobilization of stored fuels (i.e., glycogen and fat) (8).

Daily rhythms in basal blood glucose in mice under *ad-libitum* feeding exhibit high diurnal values which decrease at night (9, 10), depending both on SCN direct control, as well as on the feeding-fasting rhythm (11). Blood glucose during the activity/feeding phase is supplied mainly by diet, while during rest/fasting, by endogenous production from hepatic glycogen degradation (12). Pancreatic insulin and glucagon basal secretion, in turn, are mainly regulated by the feeding-fasting rhythm and/or changes in glycemia. Several circadian controls set increased glucose tolerance and insulin sensitivity at the light–dark transition to anticipate activity, such as islet clocks controlling peak insulin secretion (13), or the increase in glucose transporter type 4 (GLUT4) in muscle (14). In addition, the main functions of the liver are rhythmically regulated, generating a circadian control of nutrient metabolism and energy homeostasis (15). In consequence, hepatic lipid metabolism is controlled by the circadian clock (16). Some examples of this are the control of lipogenesis by the circadian clock *via* HDAC3 (17) and the regulation of adipogenesis *via* Bmal1 (18, 19).

Taking into account the circadian control over feeding/fasting, and nutrient and energy compounds metabolism, it is not surprising that, over the years, chronic circadian disruption has been hypothesized to be one of the main factors that generate metabolic alterations. At the gene level, mice lacking the Per2 gene exhibit dyslipidemia (20) and those lacking Clock have a predisposition to hyperlipidemia (21). In addition, Bmal1 in the central clock has been shown to be sufficient for controlling metabolic rhythms, being the central clock who drives the majority of rhythmicity in circulating metabolites (22).

Fan et al. showed that a chronically disrupted light protocol generated more glucose intolerance after glucose injection (23). In previous experiments in the laboratory, we found that desynchronized mice, by advancing CJL schedule, exhibit an increase in body weight (but similar caloric intake than controls), retroperitoneal and epididymal adipose tissue, adipocytes size, and circulating triglycerides (24). Moreover, light schedules inducing long-term circadian desynchronization (as constant light, dim light at night, or advancing CJL), uncouple feeding-fasting rhythm from activity/rest rhythms generating asynchronous signals to peripheral oscillators controlling energy homeostasis, leading to several metabolic disturbances (25). Thus, independent of daily caloric intake and nutrient quality, the daily timing of food consumption must be considered as a critical factor in maintaining metabolic health, by allowing circadian compartmentation of energy homeostasis.

Rhythms in both intestinal microbiota composition and their metabolome, strongly dependent on the feeding-fasting rhythm of the host, have been studied in regulating circadian nutrient and energy metabolism (26). Chronic changes of the microbiota composition (dysbiosis) are related to metabolic alterations, recognized due to a diminution in bacterial diversity, and an increment of facultative anaerobes followed by an increment in the Firmicutes/Bacteroidetes ratio. CJL schedules were used in several studies to generate circadian disruption and/or dysbiosis of rhythms in the microbiota, as reduced bacterial oscillations (27), changing the composition of jejunal and fecal microbiota (28), or shifting the Firmicutes rhythm (29). Taking this into account, under conditions of circadian desynchronization, the daily microbiota composition can be assessed as a prognostic marker of metabolic disturbances.

In the present work, we aim to continue exploring the effects of CJL on circadian metabolic homeostasis, by studying the desynchronization of feeding-fasting rhythm, daily variations in blood glucose, lipid homeostasis, and intestinal microbiota composition.

## 2. Materials and methods

### 2.1. Animals

C57BL/6J male mice (6 weeks old, purchased from Facultad de Veterinaria, Universidad Nacional de La Plata, Argentina) were allowed to acclimatize in groups of 5 individuals in polycarbonate cages for 1 week in stock rooms at animal facilities of the Universidad Nacional de Quilmes, with tap water and rodent chow (ACA Cooperación, Argentina) *ad libitum*, and wood bedding replaced every 3–4 days. Room temperature was set at 22–24°C, and 12 h light:12 h dark (LD) cycles were set with fluorescent lamps supplying 100–150 lux at cage microenvironment [lights ON/OFF at 7 a.m./19 p.m., setting zeitgeber time 12 (ZT12) at lights OFF].

### 2.2. Behavioral recordings and CJL light schedule

**Figure 1:**
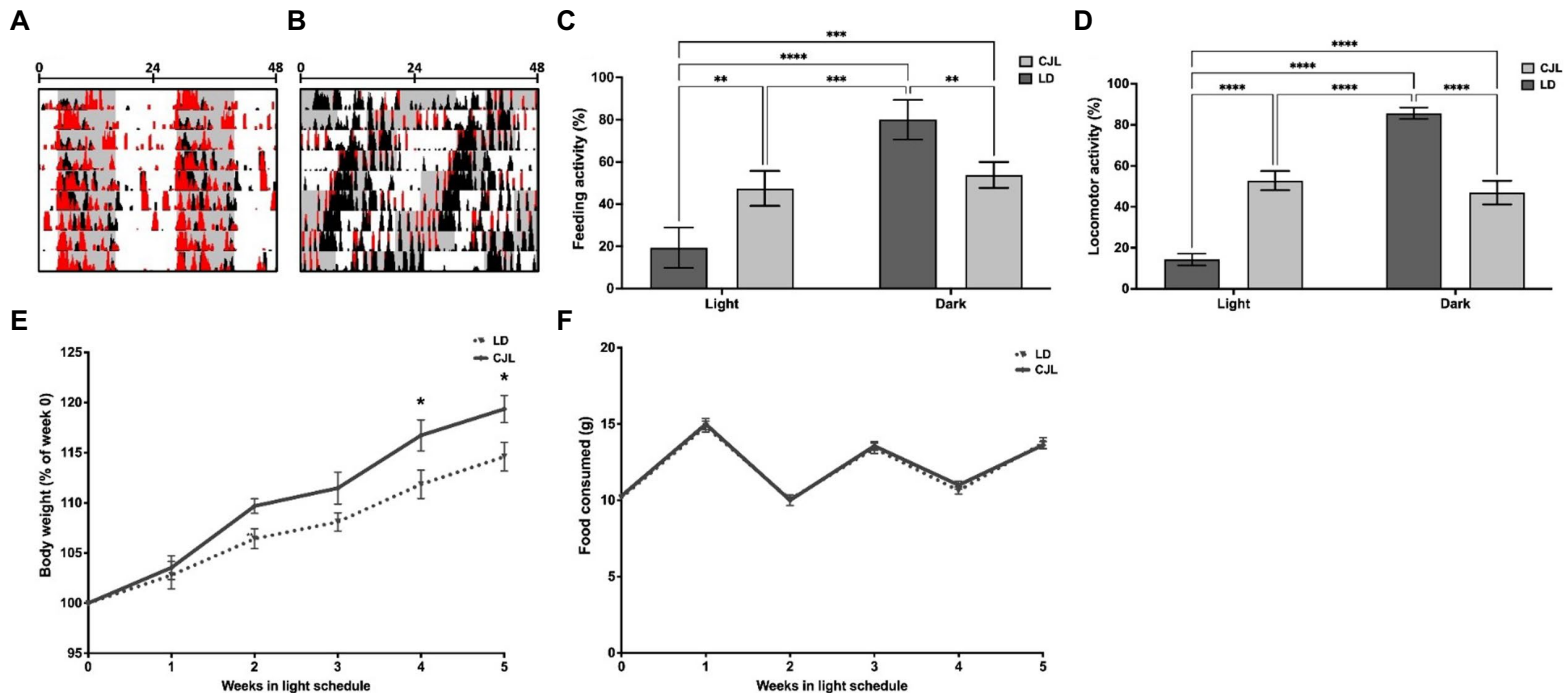
CJL induces behavioral desynchronization of general activity and feeding-fasting rhythms, together with an increase in body weight. Representative actograms of locomotor activity of mice under LD **(A)** and CJL **(B)**. Histograms in black represent general activity (black) and feeding activity (red). Mice under LD present general and feeding activity bouts at the dark phase with a period of 24 h (24 h ± 0.02 h, *n* = 4). Mice under CJL showed behavioral desynchronization with a period close to 21 h (20.97 h ± 0.02 h, *n* = 4). **(C)** light/dark feeding activity distribution (as a percentage of total) of mice under LD and CJL. Feeding activity under LD was significantly higher in the dark, while no significant difference was found between light and dark in mice under CJL; Two-way ANOVA, *p* < 0.0001, *F* = 41.27 for interaction; *p* < 0.0001, *F* = 63.21 for light schedule, *p* = 0.826, *F* = 0.050 for light/dark; *n* = 4, followed by Tukey’s multiple comparisons test: ****p < 0.0001, ***p < 0.001, **p < 0.01; *n* = 4. **(D)** Light/dark general activity distribution (as a percentage of total) of mice under LD and CJL. General activity under LD was significantly higher in the dark, while no significant difference was found between light and dark in mice under CJL; Two-way ANOVA, *p* < 0.0001, *F* = 430.2 for interaction; *p* = 0.953, *F* = 0.003 for light schedule; *p* < 0.0001, *F* = 309.1 for light/dark; *n* = 6 per group; Tukey’s multiple comparisons test: ****p < 0.0001. **(E)** Body weight increment (as a percentage of week 0) is significantly higher in mice under CJL conditions compared to LD. Repeated measures Two-way ANOVA, *p* = 0.0036, *F* = 2.36 for interaction; *p* < 0.0001, *F* = 92.54 for weeks; *p* = 0.0040, *F* = 9,948 for light schedule; *n* = 4, followed by Sidak’s multiple comparisons test: **p < 0.005, *p < 0.05; *n* = 4. **(F)** Food consumed (g) remained constant over the weeks, without differences between LD and CLJ conditions. Two-way RM ANOVA, *p* = 0.9682, *F* = 0.184 for interaction; *p* < 0.0001, *F* = 103 for weeks; *p* = 0.6697, *F* = 0.1858 for light schedule; *n* = 4, followed by Sidak’s multiple comparisons test: ns; *n* = 4.

Mice were placed individually in cages inside ventilated closets for behavioral recording, with food and water *ad-libitum* for the entire experimental protocol (except when indicated). General cage activity was monitored with passive infrared (PIR) motion sensors above the cage grid. Feeders were disposed inside the cage by a 5-cm diameter plastic cylinder supplied with chow and with a PIR on top for detecting food-access activity. PIR signals were processed and stored in a computer at 5 min bins, to obtain time series that were analyzed with the software El Temps (Universitat de Barcelona, Spain). From LD 12:12, the light schedule for advancing CJL (Casiraghi et al., 2012) was designed as follows: (1) advancing by 6 h the lights OFF, (2) followed by advancing 6 h the lights ON, (3) keeping this schedule for one entire LD 12:12, (4) repeating 1–3 (see Figure 1). Thus, the LD 12:12 cycle was repeated 2 times each week: one coincident with the LD cycle used as control (i.e., having ZT12 at 19 p.m., used for all experimental comparisons), and the following, with a 6-h advance.

### 2.3. Experimental protocol

After recording 24-h rhythms under LD 12:12 cycles for 7 days, two experimental groups were set: mice kept under LD 12:12 (control group, *n* = 14; from here, “LD”), or under CJL (*n* = 14) for 50 days, monitoring behavior, as well as measuring the weekly increase of body weight, and food intake by weighing the food remaining in feeders after 1 week. In the last 15 days of the protocol, glucose was measured in blood samples every 4 h and for 24 h, for testing daily rhythms in glycemia under *ad libitum* feeding in both CJL and LD groups. The next week, an intraperitoneal glucose tolerance test (IPGTT) was performed according to the guidelines and considerations for metabolic tolerance tests in mice (30), with previous fasting of 4 h, by delivering 2 g/kg glucose in sterile saline solution intraperitoneally, both at ZT6 and ZT18, measuring blood glucose at 0, 15, 30, 60, and 120 min, and insulin at 0, 15, 60, and 120 min post-administration.

### 2.4. Biochemical analysis

Mice were restrained for taking blood samples from the caudal venous sinus, in order to measure *ad libitum* glycemia rhythm (at ZT3, ZT6, ZT9, ZT12, and ZT24), and delivering an IPGTT at both ZT6 and ZT18 to measure glycemia (digital glucometer Contour TS, Bayer) and plasma insulinemia responses with an ELISA kit (EMINS, Thermofisher Scientific, United States) according to manufacturer’s instructions, followed by absorbance measurement at 450 nm in Cytation 5 imaging reader (Biotek Instruments, United States).

Samples at the endpoint were taken at ZT6 and ZT18 under isoflurane (5% in oxygen, 500 ml/min) anesthesia, first by bleeding out euthanized mice by cardiac puncture, for measuring total cholesterolemia (Colestat, Wiener Lab, Argentina), HDL (HDL cholesterol, Wiener Lab, Argentina) and LDL cholesterolemia (LDL cholesterol, Wiener lab, Argentina). All measurements were performed following the manufacturer’s instructions, followed by absorbance measurements (Smartspec 3,000 UV/Vis, Biorad, United States) at 505 nm for total cholesterolemia, 600 nm for HDL, and 660 nm for LDL. A subset of mice was destined for microbiota analysis, collecting a sample of cecum content according to Tong et al. (31) at ZT0 and ZT12, in order to have one time-point at the end of the feeding phase, and the other one at the end of the fasting phase.

### 2.5. Microbiota analysis

About 250 mg of cecum content homogenized in buffer GA was centrifuged, obtaining DNA from supernatants with a commercial kit (PBL, Argentina). DNA integrity and concentration were determined by spectrophotometer and electrophoresis gels. To construct the libraries V3-V4, the hypervariable region of the ribosomal 16S gene was amplified from DNA. Then a second PCR cycle was performed to add P7 and P5 adapters analyzing the amplicons by spectrophotometer and electrophoresis and then purifying them by silica columns. Each library was then sequenced by means of a Miseq 300 bp, paired-end platform (Illumina Inc., San Diego, CA, United States). Operational taxonomic units (OTUs) were then identified using DADA2 software and SILVA database and analyzed by means of R packages included in the Microbiome Analyst (32) tool. The number of OTUs reads was filtered (cutoff 4 reads, 20% prevalence, cutoff 10% of variance by interquartile range) in each sample. Then, the relative abundance (% of reads in each sample) of OTUs was obtained, and those included in Firmicutes or Bacteroidetes phyla were summed to obtain the Firmicutes/Bacteroidetes ratio.

### 2.6. Chronobiological and statistical analyses

Behavioral time series were analyzed for detecting circadian rhythms by means of actograms and Chi-square periodograms. Both locomotor activity, as well as activity at feeders, were analyzed in 24-h waveforms as follows: in control mice, by averaging activity in representative data sections of 7 consecutive days, while in mice under the CJL schedule, waveforms were obtained by averaging 4 data sections of the LD 12:12 cycles present in the schedule. The activity counts during light or dark were summed, and calculated as percentage of total activity in 24 h waveforms for comparison. Daily rhythm in glycemia was assessed by fitting mean values obtained of each ZT to the best 24-h cosine function that minimizes the squares of the residuals. Mean values obtained by averaging ZT3, ZT6, ZT9, ZT12 for light, and ZT15, ZT18, ZT21, ZT0 for dark conditions, were also compared. Statistical analyses were done using the software GraphPad Prism 7 (GraphPad Software, San Diego, CA) by previously assessing assumptions for parametric ANOVA analyses, setting *p* = 0.05 as a level for type I error.

## 3. Results

### 3.1. CJL induces desynchronization of feeding-fasting rhythms

Advancing CJL is one of the most commonly used light schedules to desynchronize the behavioral rhythms of mice. Actograms in Figures 1A,B show general (black histograms) and feeding activity (red) patterns of representative mice subjected to CJL or LD. All mice under CJL present an activity period that approximates to 21 h (20.97 h ± 0.02 h), following the global period of the light schedules, while 8 out of 14 present a simultaneous period component under relative coordination (24.11 h ± 0.19 h), showing behavioral desynchronization. General activity (24 h ± 0.02 h) and feeding activity bouts under LD were concentrated mainly in the dark (Figures 1A,C), whereas, under CJL, feeding activity also spread into the light period (Figure 1B) generating a similar amount at light and dark (Figure 1C). In addition, weight was significantly increased from the second week of the protocol in mice under CJL with respect to controls (Figure 1D), without differences in the amount of food intake (Figure 1E).

### 3.2. Glucose metabolism is altered under CJL

**Figure 2:**
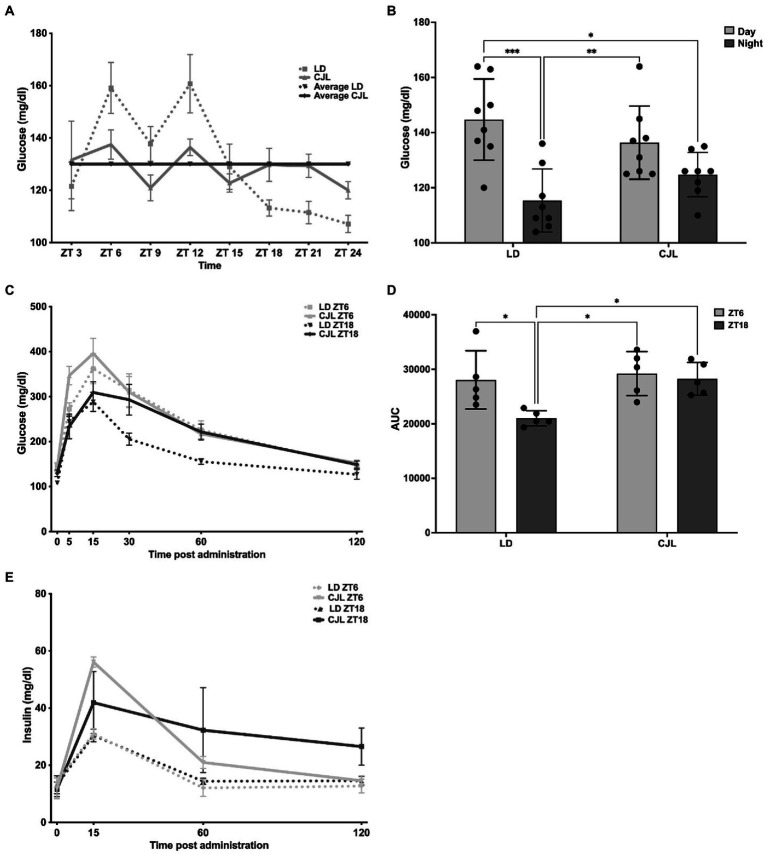
Daily glucose homeostasis is altered under CJL conditions. **(A)** Daily rhythm in blood glucose concentration (mg/dl) under *ad libitum* feeding along LD 12:12 in control mice under LD (dotted line) and under CJL (solid line). The daily mean for each group is also indicated. Two-way ANOVA, *p* = 0.0058, *F* = 3.05 for interaction; *p* < 0.0001, *F* = 6.145 for ZT; *p* = 0.682, *F* = 0.1685 for light schedule. Mean values for each ZT of control mice (*n* = 5) were significantly adjusted to a cosine wave of 24 h (Ho: amplitude = 0, *p* < 0.05), having the following parameters: MESOR: 130 mg/dl, amplitude: 34.4 mg/dl, acrophase: ZT7. **(B)** Mean values obtained by averaging ZT3, ZT6, ZT9, ZT12 for light, and ZT15, ZT18, ZT21, ZT0 for dark conditions. During the light phase, the values increase and in the dark, the values decrease for LD and CJL conditions. Two-way ANOVA, *p* = 0.0481, *F* = 4.271 for interaction; *p* < 0.0001, *F* = 22.79 for day and night; followed by Tukey’s multiple comparisons test: ***p < 0.001, **p < 0.01, *p < 0.05; *n* = 8. **(C)** Blood glucose concentration kinetics after delivering 2 g/kg (*t* = 0) intraperitoneal glucose (IPGTT) in mice under LD (dotted line) at or under CJL (solid line), both at ZT6 (gray) or ZT18 (black). Glycemia values were measured at *t* = 0, *t* = 5, *t* = 15, *t* = 30, *t* = 60, and *t* = 120 (min) after glucose administration. Significant differences were found for glycemia kinetics between LD and CJL; however, no significant differences were observed for the two ZTs. Values are expressed as mean ± SEM at each time. Three-ways ANOVA, *p* < 0.0001, *F* = 20.63 for time; *p* = 0.006, *F* = 26.75 for light schedule; *p* = 0.2861, *F* = 1.51 for ZTs; *p* = 0.28, *F* = 2.75 for time × time schedule; *p* = 0.37, *F* = 1.14 for time × ZT; *p* = 0.56, *F* = 0.39 for time schedule × ZT; *p* = 0.55, *F* = 0.72 for time × time schedule × ZT. **(D)** Value of the area under the curve (AUC) to estimate the rate of appearance/disappearance of blood glucose, as an indicator of tolerance. A lower value was observed in LD at ZT18 compared to the CJL condition. Values represented as mean ± SEM. Two-way ANOVA, *p* = 0.0871, *F* = 3.322 for interaction; *p* = 0.0227, *F* = 6.357 for light schedule; *p* = 0.0293, *F* = 5.726 for ZT; followed by Tukey’s multiple comparisons test: *p < 0.05; *n* = 5. **(E)** Blood insulin values after delivering 2 g/kg (*t* = 0) intraperitoneal glucose (IPGTT) in mice under LD (dotted line) at or under CJL (solid line), both at ZT6 (gray) or ZT18 (black). Insulin values were measured at *t* = 0, *t* = 5, *t* = 15, *t* = 30, *t* = 60, and *t* = 120 (min) after glucose administration. Values are expressed as mean ± SEM at each time.

In order to determine if chronic desynchronization by the advancing CJL schedule has an impact on glucose homeostasis, first, the 24-h daily rhythm in blood glucose *ad libitum* was measured under LD and CJL (Figure 2A). Mean values for each ZT of control mice (*n* = 5) were significantly adjusted to a cosine wave of 24 h (Ho: amplitude = 0, *p* < 0.05), having the following parameters: MESOR: 130 mg/dl, amplitude: 34.4 mg/dl, acrophase: ZT7 (higher values during light, that decreased with time at the darkness, Figure 2B). For basal glycemia measured in mice under CJL (*n* = 5), 24-h cosine fitting was not significant (amplitude = 0, *p* > 0.05), with similar values at light and dark (Figure 2B). In contrast, under the CJL condition, blood glucose values did not decrease at dark. When performing an IPGTT, blood glucose showed similar kinetics (Figure 2C) within each ZT but differs between LD and CJL groups; however, an area under the curve (AUC) test (Figure 2D) showed no significant differences at light (ZT6) for both LD and CJL, however, a significantly lower tolerance (i.e., increased AUC) was found at ZT18 under CJL. In addition, insulin release in response to IPGTT was also affected under CJL at both ZT6 and ZT18 (Figure 2E). While mice under LD increased their insulin levels from basal values to about a 3-fold change at 15 min, those under CJL showed an increased insulinemia at both ZTs. Thus, taking all these results into account, mice under CJL showed impaired daily glucose homeostasis, having increased basal nocturnal hyperglycemia *ad libitum*, both decreased glucose tolerance at night, and increased insulinemia, in response to IPGTT.

### 3.3. LDL levels are increased in mice subjected to CJL

**Figure 3:**
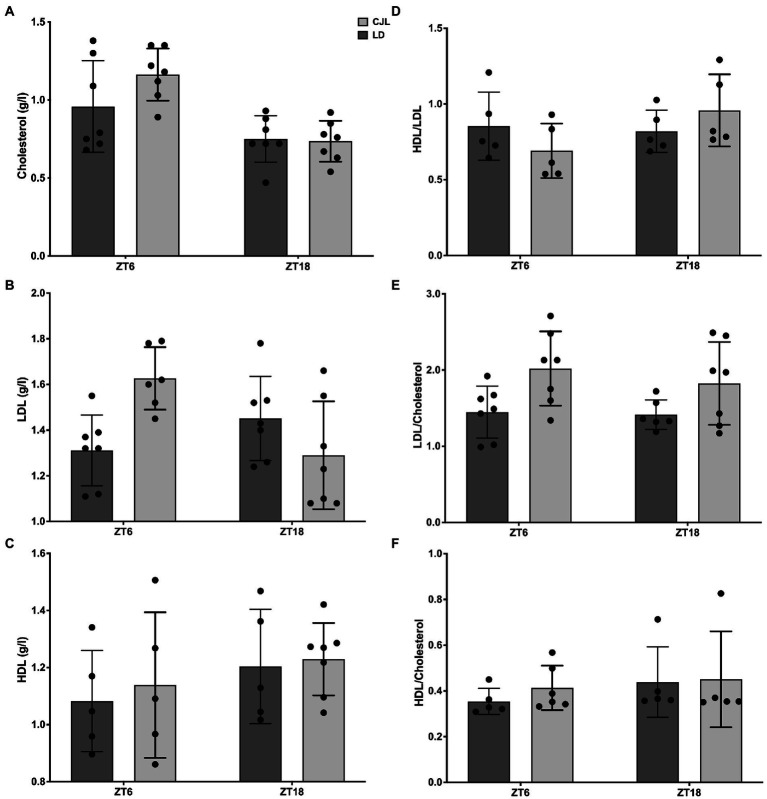
Daily changes in lipidemia (total cholesterolemia, LDL, HDL, HDL/LDL ratio, HDL/cholesterol ratio, and LDL/cholesterol ratio) in mice under LD and CJL at ZT6 and ZT18. **(A)** Total cholesterol (g/l) two-Way ANOVA, *p* = 0.1533, *F* = 2.174 for interaction; *p* = 0.0003, *F* = 18.39 for ZT; *p* = 0.2122, *F* = 1.643 for light schedule; followed by Sidak’s multiple comparisons test: ns; *n* = 7. **(B)** LDL (g/l) two-way ANOVA: *p* = 0.002, *F* = 11.33 for interaction; *p* = 0.178, *F* = 1.929 for ZT; *p* = 0.288, *F* = 1.180 for light schedule. **(C)** HDL (g/l), two-way ANOVA: ns. **(D)** HDL/LDL ratio, two-way ANOVA: ns. **(E)** LDL/Cholesterol ratio, two-way ANOVA, *p* = 0.6226, *F* = 0.248 for interaction; *p* = 0.4876, *F* = 0.4977 for ZT; *p* = 0.0061, *F* = 9.106 for light schedule; followed by Tukey’s multiple comparisons test: ns; *n* = 7. **(F)** HDL/Cholesterol ratio, two-way ANOVA: ns.

Lipidemia was measured in order to analyze the impact of CJL on the daily homeostasis of lipid metabolism. While total cholesterol, LDL, HDL, HDL/LDL, and HDL/cholesterol ratio did not change with respect to controls under LD (Figures 3A–D,F), two-way ANOVA shows that LDL/cholesterol ratio significantly increased in mice under CJL, both at ZT6 and ZT18 (Figure 3E), but no significant differences were found between ZT6 and ZT18 for either LD or CJL, indicating that time of day does not have an impact on LDL/cholesterol levels, as opposed to the light schedule.

### 3.4. Microbiota composition is altered in CJL

**Figure 4:**
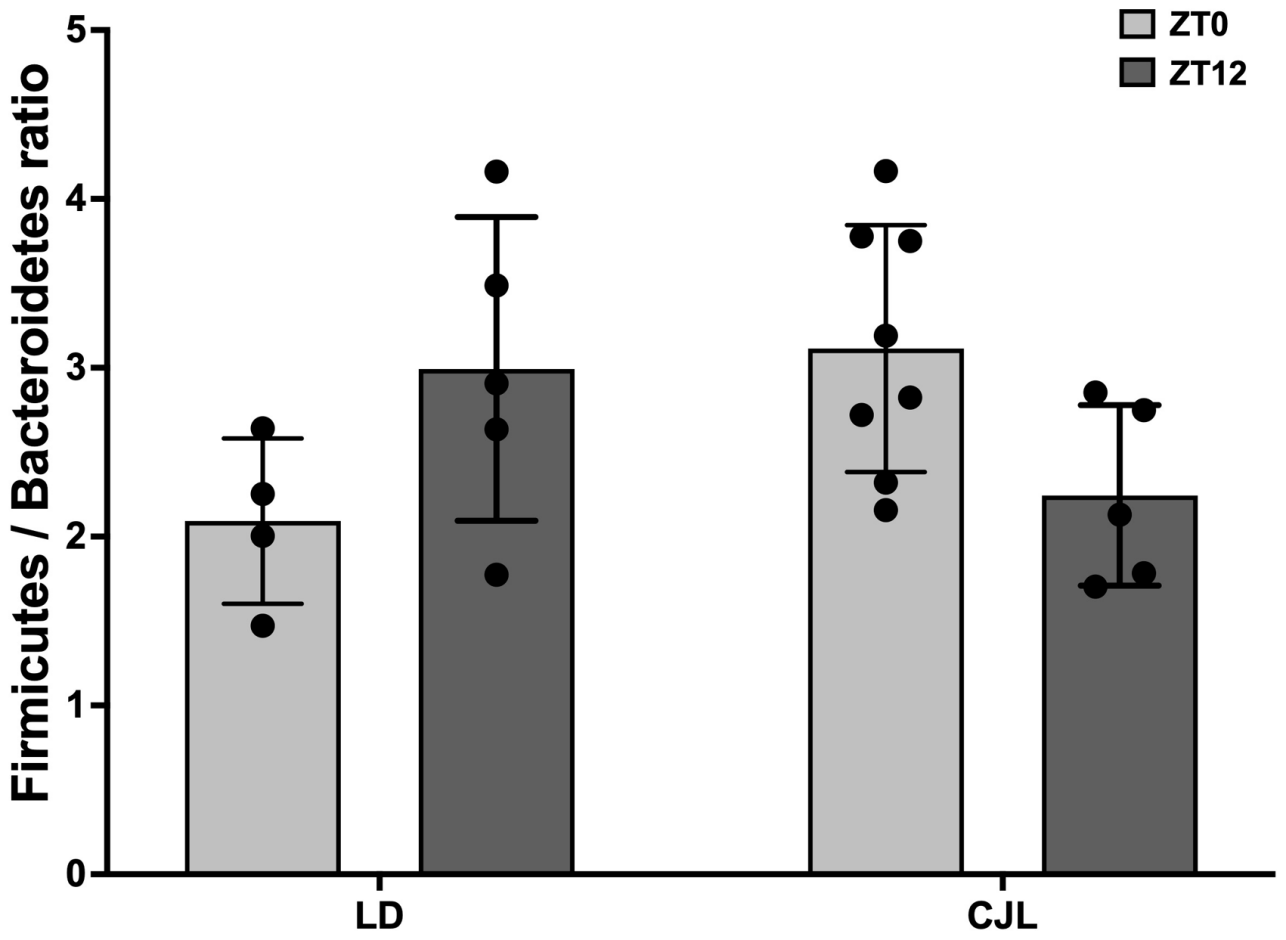
Daily changes in gut microbiota (Firmicutes/Bacteroidetes ratio) composition for mice under LD and JCL at ZT0 and ZT12. Two-Way ANOVA, *p* = 0.0102, *F* = 8.235 for interaction; *p* = 0.6646, *F* = 0.1943 for light schedule; *p* = 0.9592, *F* = 0.0026 for ZT; followed by Tukey’s multiple comparisons test: ns; *n* = 8.

First, samples of cecum content were collected from mice under both light schedules at ZT0 and ZT12, and microbiome composition was determined by performing sequencing of the samples, obtaining the relative abundance of the different Phylum (Supplementary Figure 1A) and Families (Supplementary Figure 1B) present on each sample. To determine differences between these compositions, the analysis was centered on determining the difference between Firmicutes and Bacteroidetes relative abundances (% of reads in each sample), obtaining the Firmicutes/Bacteroidetes ratio at ZT0 and ZT12, in order to obtain daily markers of dysbiosis. This ratio was changed at both light and dark conditions depending on light schedules (LD vs. CJL; i.e., interaction is a significant factor in the ANOVA, Figure 4). This indicates a misalignment of the microbiota populations with the time of day, due to the chronic desynchronization of the feeding/fasting rhythm.

## 4. Discussion

In the present work, we studied the effects of chronic CJL on the homeostasis of energy, metabolism, and weight. Mice present desynchronization of behavioral activity and feeding activity, resulting in weight gain by feeding a similar daily amount of food to controls. In addition, *ad libitum* glycemia rhythm was abolished in CJL-subjected mice and an IPGTT shows glucose intolerance in dark, with increased insulin release at both light and dark periods. When lipids were analyzed, we found that LDL was increased, but no changes in HDL were observed.

When set in opposite phases, and correctly aligned with peripheral oscillators, activity/feeding and rest/fasting cycles act as strong zeitgebers for the circadian compartmentation of the metabolism of energy compounds. In this work, chronic advances of the LD cycle induced behavioral desynchronization with several metabolic disturbances, generating increased weight gain, with a similar amount of food intake [as observed in other studies by the lab (24) or other groups (27)]. Behaviorally desynchronized mice under CJL show feeding activity still coincident with general activity (see actogram in Figure 1). Also, in mice under CJL, daily caloric intake was not different from controls but distributed similarly at light and dark.

While mice under LD decreased *ad libitum* glycemia at dark after activity onset at ZT12, desynchronization under CJL abolished this daily variation, keeping similar blood glucose at light and dark (see Figures 2A,B). Importantly, tolerance to intraperitoneal glucose was decreased at dark under CJL (Figures 2C,D), as has already been shown for other desynchronization protocols (23), despite increased insulin secretion (Figure 2E). These results indicate an impediment to managing glucose output and could explain the nocturnal hyperglycemia observed *ad libitum*. It is well described that glucose uptake by the liver, adipose, and muscle tissues increases during the night in mice, due to increased glucose tolerance and insulin sensitivity (12), to supply energy for activity. In addition, glucose transporter 4 (GLUT 4) has already been shown to be controlled by the circadian clock in muscle in mice (33) and in adipose tissue in humans (34). Thus, circadian chronic desynchronization may have an impact on GLUT4 levels, affecting consequently glucose tolerance in CJL mice.

In LD mice, intraperitoneal glucose elicited similar insulin secretion (about a 3-fold change from basal levels at 15 min) at ZT6 and ZT18, as observed in other studies (12). However, resistance to insulin actions follows a circadian rhythm, being higher at light during rest (35). Indeed, circadian disruption by advancing CJL schedule increased fasting glycemia with loss of hypothalamic insulin sensitivity (decrease of phospho Akt and insulin receptor substrate 1) (36) and delayed pancreatic genes controlling sensitivity (37). The increased glucose output at night in synchronized mice, due to increased disposal in tissues, allows daily glucose usage and/or storage according to the circadian demand for energy.

One of the limitations of the current study is that the GTT was performed with ip administration of glucose, which might result in possible artifacts derived from a less physiological route than oral administration. In addition to this, some of the effects found in this test might be limited by the small sample size. Further experiments will be conducted with oral administration and a greater number of mice. Finally, despite measuring insulin after a GTT is an accepted method, an insulin tolerance test would better measure insulin resistance. Thus, insulin tolerance tests will be conducted in the future to complete insulin resistance results.

The control of the SCN clock on daily glucose metabolism was previously assessed in SCN-ablated mice, showing a lack of rhythms in glucose tolerance and insulin sensitivity (38), oxygen consumption, and insulin resistance (39). Several genetic models (mice with Clock mutation and Bmal1 deficiency), showed disrupted circadian control of glucose, by altered gluconeogenesis (9, 40). However, these effects were assessed downstream of the SCN without a 24-h feeding-fasting rhythm as a strong feedback zeitgeber. Here, the desynchronization of mice with a functional circadian system by advancing CJL allowed the assessment of the effects of altered daily timing of feeding-fasting on the daily glucose homeostasis.

Circadian desynchronization of behavior by CJL, with increased feeding at light, generates disruption of lipid metabolism at LDL cholesterolemia, while HDL/cholesterolemia remains unaltered (Figure 3). Although a different protocol, daytime feeding was previously used to desynchronize peripheral oscillators from feeding-fasting, which increases total cholesterol due to the increased expression of cholesterol synthetic genes (41) or due to dysregulation in bile acids synthesis (42). Dissecting the central and/or peripheral control of functions at metabolic tissues depends on depict complex interactions. Oscillator in the liver is central in this network, in the circadian control at the transcriptome level of other metabolic tissues, as white adipose, upon daytime feeding (43), and its full circadian function is set by other peripheral signals dependent on photic cycle (44).

Nevertheless, LDL and HDL have not been studied under desynchronization by light schedules. This work shows that both total and HDL cholesterolemia did not change, but LDL does increase under CJL. Low-density lipoprotein receptor (LDLR) is regulated by the circadian clock *via* the complex Clock/Bmal1 (45) In addition, fatty acid binding protein 4 (FABP4,) one of the main lipid-binding proteins in adipose tissue and liver, has been shown to have a circadian expression in both tissues (46) and it is overexpressed in the liver in the contexts of morbid obesity and insulin resistance (47). It is possible that CJL is changing both levels of LDLR and FABP4, contributing to generating the differences observed in weight gain and LDL/cholesterol ratio levels. This remains to be established in further experiments. Finally, impairments in glucose uptake/disposal leading to chronic hyperglycemia and hyperinsulinemia, as observed here due to CJL, might lead to an increased usage of fatty acids from triglycerides stores in the liver and adipose tissue, as observed in previous studies in the laboratory (24). Both elevated triglyceridemia and LDL, as well as lower HDL, are biomarkers of metabolic syndrome and type 2 diabetes (48). Although this CJL mice model generates clear dysregulation of glucose homeostasis, it was not enough with respect to lipid metabolism to characterize those alterations.

Fecal Firmicutes/Bacteroidetes ratio shows a circadian (49) and daily (50) oscillation. While the abundance of Firmicutes increases during the activity/feeding period, Bacteroidetes increase during the rest/fasting period (51), due to their ability to degrade glycans of the mucosal layer cells (52). Here, the Firmicutes/Bacteroidetes ratio observed in LD was changed under CJL. Increased postprandial glucose transport at enteroendocrine L-cells, together with short-chain fatty acids (SCFA) metabolized by Firmicutes, increased both secretions of glucagon-like peptide 1 (GLP1), which in turn stimulates insulin secretion (53) as well as insulin signaling in adipocytes and glucose uptake by the tissues (54). In addition, GLP1 sensitivity has a diurnal pattern that, in turn, depends on the intestinal clock gene expression and the presence of Rumninococcaceae and Lachnospiraceae gut bacteria (55). This so-called incretin effect supplies additional circadian controls for blood glucose and lipid homeostasis (56, 57). Decreased Firmicutes/Bacteroidetes ratio, associated with decreased SCFA-GLP1-insulin signaling and glucose changes observed here, remains to be determined.

To conclude, chronic desynchronization generates evident alterations in feeding fasting rhythm and in glucose homeostasis, with nocturnal hyperglycemia related to decreased nocturnal response to glucose, hyperinsulinemia, and increased levels of LDL. Thus, it remains to be established if this model can be assessed for the development of insulin resistance by studying GLUT4 transport at the liver or dyslipidemia by studying markers of lipid export (e.g., FABP2-4), both at liver and adipose tissues.

## Data availability statement

The original contributions presented in the study are publicly available. This data can be found at: https://www.ebi.ac.uk/ena/browser/view/PRJEB60019.

## Ethics statement

The animal study was reviewed and approved by Institutional Animal Care and Use Comitee, Universidad Nacional de Quilmes.

## Author contributions

LT and ML were equal contributors and performed the experiments and wrote the manuscript. IA and RR served as technical assistance. CB and MB performed the microbiota analysis. DG served as experimental advisor and wrote the manuscript. SP and JC were both major and equal contributors in writing the manuscript, directing the study, designing experiments, and processing and interpreting data. All authors contributed to the article and approved the submitted version.

## Funding

This work was supported by grants from Universidad Nacional de Quilmes (PUNQ 1397/16) and by Consejo Nacional de Investigaciones Científicas y Técnicas (CONICET, PICT 1745-2017) from Argentina. Study design, sample collection, data analysis and interpretation, and writing of the manuscript were performed by the authors with no participation of the funding agencies.

## Conflict of interest

The authors declare that the research was conducted in the absence of any commercial or financial relationships that could be construed as a potential conflict of interest.

## Publisher’s note

All claims expressed in this article are solely those of the authors and do not necessarily represent those of their affiliated organizations, or those of the publisher, the editors and the reviewers. Any product that may be evaluated in this article, or claim that may be made by its manufacturer, is not guaranteed or endorsed by the publisher.
