# Supplementary material for: Chronic circadian desynchronization of feeding-fasting rhythm generates alterations in daily glycemia, LDL cholesterolemia and microbiota composition in mice
Source: Front Nutr. 2023 Apr 14;10:1154647. doi: 10.3389/fnut.2023.1154647 (PMC10145162; doi:10.3389/fnut.2023.1154647)
Supplement: Supplementary file 1 [file Data_Sheet_1.PDF]

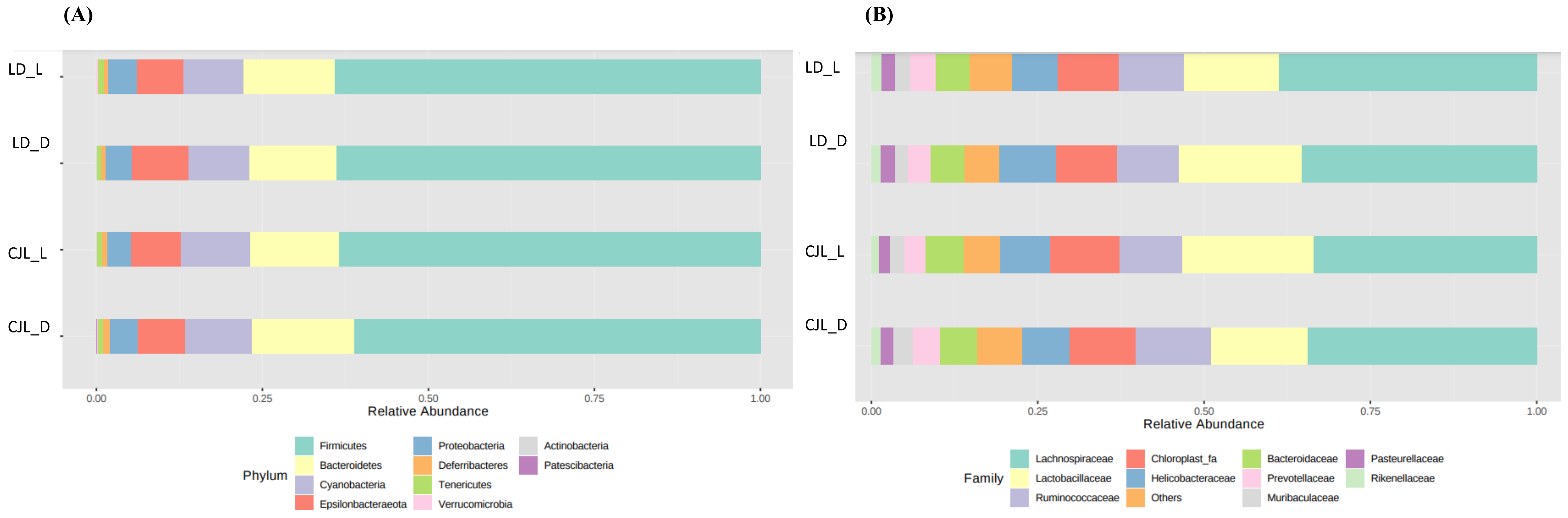

Supplementary Figure 1. Microbiota composition (relative abundance) for each experimental group (LD light, LD dark, CJL light and CJL dark) for (A) Phylum and (B) Family. OTUs with less than 10 reads are included in the group "Others".
